# Design and implementation of integrated solid wastes management pattern in industrial zones, case study of Shahroud, Iran

**DOI:** 10.1186/2052-336X-12-32

**Published:** 2014-01-14

**Authors:** Nazemi Saeid, Aliakbar Roudbari, Kamyar Yaghmaeian

**Affiliations:** 1 Department of Environmental Health Engineering, School of Public Health, Shahroud University of Medical Sciences, Shahroud, Iran; 2 Center for Health-Related Social and Behavioral Sciences research, Shahroud University of Medical Sciences, Shahroud, Iran; 3 Department of Environmental Health Engineering, School of Public Health and Institute for Environmental Researches, Tehran University of Medical Sciences, Tehran, Iran

**Keywords:** Solid wastes, Integrated management, Industrial zone

## Abstract

**Background:**

The aim of the study was to design and implementation of integrated solid wastes management pattern in Shahroud industrial zone, evaluates the results and determine possible performance problems. This cross - sectional study was carried out for 4 years in Shahroud industrial zone and the implementation process included:1- Qualitative and quantitative analysis of all solid waste generated in the city, 2- determine the current state of solid waste management in the zone and to identify programs conducted, 3- Design and implementation of integrated solid wastes management pattern including design and implementation of training programs, laws, penalties and incentives and explain and implement programs for all factories and 4- The monitoring of the implementation process and determine the results.

**Results:**

Annually, 1,728 tons of solid wastes generated in the town including 1603 tons of industrial wastes and 125 tons of municipal wastes. By implementing this pattern, the two separated systems of collection and recycling of domestic and industrial wastes was launched in this zone. Also consistent with the goals, the amount of solid wastes generated and disposed in 2009 was 51.5 and 28.6 kg per 100 million Rials production, respectively.

**Conclusion:**

Results showed that implementation of pattern of separated collection, training programs, capacity building, providing technical services, completing chain of industries and strengthening the cooperation between industrial estate management and industrial units could greatly reduce the waste management problems.

## Introduction

Iranian industrial facilities generate and dispose of approximately 1.1 billion tons of industrial solid wastes each year from 17 different industry groups such as organic chemicals, inorganic chemicals, primary iron and steel, plastics and resin manufacturing, stone, clay, glass and concrete, pulp and paper, food and kindred products [1]. Estimates indicated that the amounts of industrial wastes increased by 6.8% while at the same time municipal wastes has increased by 9.8% per year in Iran [2]. If not properly managed, the accumulation of industrial wastes within the industrial zones can lead to environmental damages, as well as increased safety problems and health-care costs [3]. One of the significant challenges facing industrial zone managers is how to minimize the negative impacts of solid wastes while still attempting to promote rapid industrial development [4,5]. Studies show that research conducted in this area in our country just focus on the current status of the production and handling of this material and there is no enforcement mechanism to improve the management of these materials. As an example, study conducted by Binavapour on industrial wastes in Hamadan industrial zone showed that hazardous waste minimization and separation is performed in this zone but there is no special compartment for collecting industrial wastes [6]. Study conducted by Mesgarof on industrial wastes in Kermanshah industrial zone showed that 76.32% of industries kept their wastes in dumping site, 80% of industrial wastes are recycled and 11% of industries disposed their wastes as unsanitary dumping. They concluded that the implementation of appropriate systems to manage solid wastes in the zone is necessary [7]. In a study of Sanandaj Industrial zone, Ghavami showed that the current status of solid waste management is not in accordance with the principles of environmental and long-term storage of waste at zone may cause adverse consequences on the environment. However, Bamani’s study was the only study in which it was proposed to have a database of hazardous wastes. However; the study did not provide the solution to solve the problem of all industrial wastes generated in industrial zone [8]. Many studies have been done at world in the field of industrial waste management. Hogland has provided a model for solid waste management is based on three pillars: economic, energy and environmental impacts. They have concluded that this model can have great advantages with small changes in industrial processes but carbon dioxide emissions will increase slightly [9,10]. To improve the management of industrial wastes in Poland, Malgorzata suggested that Europe Union rules replace Industrial Waste Management Rules in Poland, Industrial production cycle of a product to be considered separately, Industrial waste management budget increase and local industries encourage following solid wastes sustainable management patterns [11]. Integrated solid wastes management pattern is a method can integrate industrial development programs with environmental rules and prevent adverse environmental impacts associated with solid wastes without interrupting the industrial development. The aim of the study was to design and implementation of integrated solid wastes management pattern in Shahroud industrial zone; evaluate the results and determine possible performance problems.

**Figure 1:**
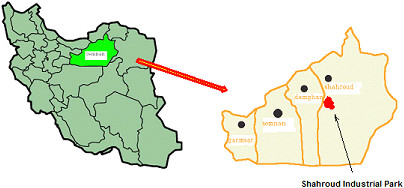
Map of Iran and Shahroud city.

Shahroud industrial Zone (SIZ) located in Semnan province in north east of Iran (Figure 1) which is approximately 5 km from the city center of Shahroud. It has a planned area of nearly 410 hectare and at the end of 2011, approximately, 1235 workers or managers travel every day between the city of Shahroud and the zone. Through almost one decade of development, SIZ has established seven groups of industries: electronic (7 units), foodstuffs (9 units), metal (12 units), chemicals (9 units), nonmetal (6 units), textile (3 units) and Cellulose industry (3 units). Iran industrial estates have the responsibility for managing SIZ and set up an administrative agency to oversee the daily administration of the zone [12]. This agency is called the Semnan industrial estates office (SIEO); it is comprised of a CEO, two deputies and four departments [13]. The main functions of this office include enforcing national laws and regional regulations, monitoring environmental protection, levying tax, stipulating economic and social development policies, and managing public financial resources [14].

## Materials and methods

This cross-sectional study was carried out for 4 years in Shahroud industrial zone and the implementation process included: 1- Qualitative and quantitative analysis of all solid wastes generated in the zone according to the guidelines listed in the book of industrial wastes management, theory and practices By John Pitchel.

2- Determine the current state of solid wastes management in the zone and to identify programs conducted. For this purpose, a team of experts under the auspices of academic specialists were trained. They collected detailed information on how each plant’s solid wastes management using various methods such as interviews with industrial zone manager, Fill out the questionnaire and in-place measurement of parameters. Table 1 shows the questionnaire.

**Table 1:** Questionnaire used for data gathering

	**Name of industry**	**Area of activity**	**Number of staff**
	**Name of interviewee and responsibility**	**Place of interview**	**Date**
1	Does the factory have environmental health engineer or industrial health supervisor?		
2	What training courses has he participated from employment?		
3	Has the manager of industry zone held special training courses and programs for your environmental health engineer or industrial health supervisor?		
4	Do you get paid to manager of industry zone for the cost of wastes management services? IF yes, how much is this annually?		
5	Name and amount of raw materials needed daily?		
6	Name and amount of daily production?		
7	Name and amount of daily solid wastes production?		
8	Do you collect your solid wastes separately?		
9	If the answer to question eight is positive, how?		
10	How much of the solid wastes produced in factory will be sold to recycler individuals or groups or is delivered to other industries?		
11	How much of the solid waste generated in factory is burned? Where?		
12	How much of the solid waste generated in factory is buried? Where and how (landfill, dumping, with or without pay)?		
13	What are the future plans to improve solid waste management in factory?		

3- Design and implementation of integrated management pattern including design and implementation of training programs, laws, penalties and incentives and explain and implement programs for all factories. The new industrial solid wastes management plan is composed of four activities include: a. Development of a solid wastes management system that serves the zone’s long-term strategy to become a center of manufacturing in the North east of Iran (Nor against it) [15], b. Establish a system of environmental information on ways to reduce the production and reuse and recycling of solid wastes [16], c. Encouraging industry to use new strategies such as environmental management, cleaner production, life cycle management which tries to reduce the lifetime environmental impact of a product and also get ISO14001 certification [17], d. Construction of new facilities for separating and classifying ecological industrial solid wastes generated in the zone [18]. In order to implement this new system, industrial zone manager attempted to develop indicators to measure the achievement or non-achievement of the objectives of system. The indicators are listed in Table 2 are: The amount of solid wastes generated per Gross domestic product (GDP), the amount of solid wastes collected and disposed per Gross domestic product, the amount of solid wastes recycled, the amount of paper and plastic recycled and the amount of hazardous wastes disposed safely. These indicators are created using analyzing patterns of solid wastes management in developing countries, interview with experts in the field of industrial wastes management in Iran (Ad-Hoc method), determining the current level of the these indicators in the industrial zone, interview with managers of factories located in zone, Considering the zone technical potential and considering the principle of “Indicators should be consistent with reality”. In general, the purpose of this pattern is reducing the amount of solid wastes generated half the current value and increasing the recycling rate 2.5 times the current amount over a 10 year period.

**Table 2:** Goals of solid waste management in SIZ

**Indicators**	**2009 (Benchmark) (g/year)**	**Start year (2010)**	**2014 (mid-term)**	**2019 (long-term)**
Amount of solid wastes production per GDP^b^ (kg/10^8^Rials^a^)	66.68	63.5	47.6	40.1
Amount of solid wastes disposal per GDP (kg/10^8^Rials)	35.78	33.5	22.1	17.4
Rate of industrial solid wastes reclamation (%)	26	30	50	80
Recycling rate of wasted paper and plastic (%)	31	35	55	85
Rate of safe disposal for hazardous wastes (%)	100	100	100	100

a. Rial is Iran’s official currency. According to recent exchange rate, 1US$D = 12560 Rial.

b. Gross Domestic Product.

SIZ has tried to reach the objectives of integrated solid waste management through the “Waste Management Hierarchy” strategy, namely, reduction, reuse and recycle, incineration, and land disposal [19]. This strategy was implemented at two levels (factory and industrial zone).

### A. At each factory level

The main activities of each factory were: a. introducing and promoting the idea of cleaner production with technical support, organizing training courses and impose fines and tax incentives, b. Establish a fund to support cleaner production, c. Implementation programs related to cleaner production such as auditing, technical updates and special training programs for managers and employees, d. Implementation of source separation, life cycle assessment and emission treatment, e. Developing incentive, controlling and selling of products mechanisms, f. Construction of facilities necessary to buy expensive wastes such as wasted paper, metals and plastics in zone and granting the privilege to collect the wastes from all factories in zone and g. collection and transport of hazardous wastes such as adhesives, solvents, flammable and toxic materials to hazardous wastes treatment facility located within the zone.

### B. At industrial zone level (in general)

**Figure 2:**
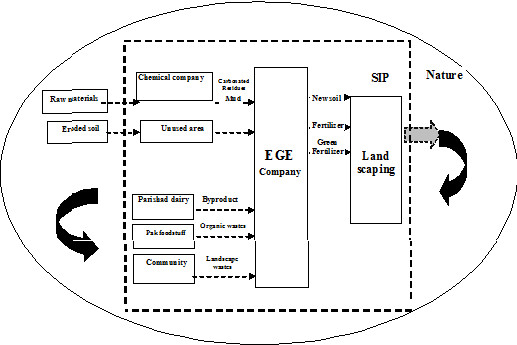
Components of cluster of cluster of East environmental company with Parishad dairy industry.

**Figure 3:**
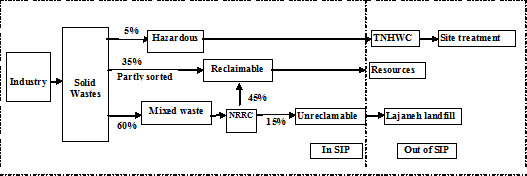
A planned sorting, collecting and recycling system for industrial waste in SIZ.

**Figure 4:**
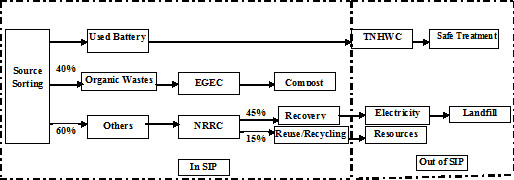
A planned sorting, collecting and recycling system for municipal waste in SIZ.

The main activities at industrial zone level (in general) were: a. creating a cooperative industrial ecosystem by encouraging by-products exchange among factories; changing factories cycle in zone from producers and consumers into producers, consumers, scavengers and decomposers [20,21], b. Creating a website to share information and encourage by-products exchange and creating clusters of industrial cooperation between factories such as cluster of East Environmental company with Parishad dairy industry. Figure 2 shows the components of this cluster. c. Legislation with the goal of having all activities comply with environmental norms such as enforcement of in-source separation and reduction pattern of industrial and municipal solid wastes due to the future expansion of zone and anticipated future increase in solid wastes (Figures 3 and 4), Special incentive policies for scavengers and decomposers factories such as providing information about the zone wastes, reduce taxes and subsidies to offset operating costs, implementation of the principle of “Pay off the cost of pollution”, granting tax breaks and economic incentives, supporting research and joint activities in the field of solid wastes management between tenant factories and other organizations and academic units and also adopting measures to deal with accidents and emergencies (called the possible use of the site) and d. Providing technical assistance and training for industries due to the limited environmental management programs, especially for managers and strengthen the relationship between zone and Shahroud city solid wastes management programs because there are some shared facilities for treatment and final disposal of solid wastes.

4- The monitoring of the implementation process and determine the results and trying to fix possible problems. For assessing the effectiveness or non effectiveness of this model to reduce or eliminate the problem of solid waste management, zone manager was determined the level of achievement to goals (Table 2) at the end of each year.

Dioxins in ambient air were collected in the glass fiber filters and the polyurethane foams. The samples to be determined were extracted and prepared, and then dioxins were quantified by the HRGC-HSMS.

## Results and discussion

### A. Status of solid wastes management in the industrial zone before implementing integrated solid wastes management pattern

The total amount of solid wastes produced in SIZ in 2009 was 1728 tons, including 1603 tons of industrial waste and 125 tons of municipal wastes [1]. Tables 3 and 4 present the quantity of solid waste generated in SIZ in 2009. As Table 4 shows organic matter, paper, paperboard and demolition and construction wastes constitute over 80% of total wastes.

**Table 3:** Solid wastes quantity generated in SIZ in 2009

**Type of solid wastes**	**Production amount (tons)**	**Percentage (%)**
Industrial wastes:		
Sent to landfill	626.57	36.26
Collected by scavengers	-	-
Reused/recycled	884.75	51.2
Hazardous	91.58	5.3
Municipal wastes	125.1	7.24
Total	1728	100

**Table 4:** Solid wastes composition in SIZ in 2009

**Waste type**	**Total quantity in 2011 (ton/year)**	**Percentage of the total wastes (%)**
Organic wastes	494.2	28.6
Wasted wood	32.83	1.9
Sludge	24.19	1.4
Wasted metal	53.57	3.1
Wasted paper	257.47	14.9
Wasted plastics	269.57	15.6
Leather	20.74	1.2
Textile	22.47	1.3
Hazardous wastes	91.58	5.3
Wasted oil	112.32	6.5
Wasted solvent	139.97	8.1
Construction wastes	112.3	6.5
Others	96.77	5.6

Although SIZ is still in the early stage of managing their solid wastes, several solid waste treatment facilities have been established, including an energy-recovery incinerator and a landfill. The SIZ Solid Waste Incinerator was built in 2010. It employs Finland technology and can meet the EU emissions standard. One indicator is that the dioxin concentration of its emissions is less than 10^-10^ g/m^3^, while the current Iran standard is 10^-9^ g/m^3^. The local landfill, namely, Lajaneh Landfill, was established in 2009 and can accept up to 100 tons of solid wastes per day. This facility locates outside SIZ, but can provide services to SIZ factories and industries. This landfill employs semi-aerobic landfill technology and implements many of the practices, such as leachate and gas collection. However, longer distance between SIZ and the landfill (25.5 km) have impeded some SIZ factories from sending their wastes to the landfill. Some factories especially the national firms such as Moghan Wire and Cable, Turbo Generator and Wasegh Forg Electric factories have already established their own management systems and programs on managing solid wastes include ISO 14001 certification, cleaner production and waste minimization initiatives. For instance, the Moghan wire and cable, has successfully refined copper residue from the wire coating process However, such practices are still few and most factories have not recognized the significance of minimizing their solid wastes [2].

### B. The results of the implementation of the integrated solid wastes management pattern in this zone

Table 5 shows the results of the implementation of the integrated solid wastes management pattern in this zone.

**Table 5:** Results of implementation of the integrated solid wastes management pattern in this zone

**Goal**	**2010**		**2011**		**2012**	
	**Goal**	**Level achieved**	**Goal**	**Level achieved**	**Goal**	**Level achieved**
Amount of solid wastes production per GDP (kg/10^8^Rials)	63.5	64.2	60.32	59.31	57.14	56.10
Amount of solid wastes disposal per GDP (kg/10^8^Rials)	33.5	34.2	31.22	30.10	28.94	27.54
Rate of industrial solid wastes reclamation (%)	30	27.81	34	32.84	38	36.12
Recycling rate of wasted paper and plastic (%)	35	34.1	39	37.85	43	41.69
Rate of safe disposal for hazardous wastes (%)	100	100	100	100	100	100

As Table 5 shows amount of solid wastes generated from 66.68 kg per 100 million Rials production in 2009 has been reduced to 64.2, 59.31 and 56.10 in 2010, 2011 and 2012, respectively that means, more than 98 percent of the goal has been realized. Also, amount of solid waste disposed from 35.87 kg per 100 million Rials production in 2009 has been reduced to 34.2, 30.10 and 27.54 in 2010, 2011 and 2012, respectively that means, more than 96.5 percent of the goal has been realized. Based on the data presented in Table 5, Recycling rate of wasted paper and plastic from 26 percent in 2009 has been increased to 27.81, 32.84 and 36.12 percent in 2010, 2011 and 2012, respectively that means, more than 97.2 percent of the goal has been realized. Also Recycling rate of wasted paper and plastic from 31 percent in 2009 has been increased to 34.1, 37.85 and 41.69 percent in 2010, 2011 and 2012, respectively that means, more than 96.6 percent of the goal has been realized.

The results showed that strategies taken in the zone has been largely successful in achieving the goals of integrated solid wastes management pattern and led to the reduction of wastes production and increase recycling of valuable components of solid wastes. however, there is still a gap of 2 to 4% of the targeted amount because of unfamiliarity of factories manager with the pattern, lack of information about it and lack of environmental manager in some of these industries in order to careful monitoring of the implementation of this pattern. Amount of solid wastes production per GDP (goal and level achieved) in the study were 5 and 4.7 percent, respectively but the level achieves by Pires in industrial zone in France was 8 percent. The reasons for this difference are different technology level and the current state of solid waste management in the two countries [22,23]. Amount of solid wastes disposal per GDP (goal and level achieved) in the study were 4 and 3.87 percent, respectively but the level achieves by Mbuligwe in industrial zone in Tanzania was 2.5 percent. The reasons for this difference are different technology level and more accurate monitoring [19]. Rate of industrial solid wastes reclamation (goal and level achieved) in the study were 4 and 3.87 percent, respectively but the level achieves by Pires and Mbuligwe 6.8 and 2.2 percent, respectively. Also, Recycling rate of wasted paper and plastic (goal and level achieved) in the study were 4 and 3.82 percent, respectively but the level achieves by Pires and Mbuligwe 7.9 and 2.3 percent, respectively. The reasons for this difference are different technology level and more accurate monitoring [24].

## Conclusion

This study showed that the pattern of integrated solid waste management is useful tool for strategic planning and management of solid wastes in an industrial zone because it will creates relations between producers, consumers, scavengers and decomposers such as a natural ecosystem among the factories located in zone to ensure the survival and viability of the zone and in its factories. This pattern is economical and valuable because solid waste back into the production cycle again. Also, it leads to the conservation of natural resources and reduce solid wastes disposal rate and cost of production.

## Competing interests

The authors declare that they have no competing interests.

## Authors’ contributions

This study is a part of a research project. The study was directed by Dr. A.A.R who is the corresponding author and made the final preparation of article. Engineer S.N was engaged in sample preparations and laboratory work. Dr. K.Y helped on analytical consulting. The overall implementation of this study including the design, sample collection and preparations, laboratory experiments, data analysis, and manuscript preparation was performed by the corresponding author and the above team. All the authors have made extensive contribution into the review and finalization of this manuscript. All authors have read and approved the final manuscript.
